# Pamphlets Received

**Published:** 1883-10-20

**Authors:** 


### PAMPHLETS RECEIVED.

Third Annual Report of the Astronomer in Charge of the Horological and Thermometric Bureaus in the Observatory of Yale College. 1882–1883. Presented to the Director of the Observatory, June 18, 1883, by Leonard Waldo. New Haven: Tuttle, Morehouse & Taylor, Printers. 1883.

Diagnosis of Ovarian Tumors. Lectures Delivered by Edw. Borck, A.M. M.D., Professor of Surgery, etc , etc.

The New York Post-Graduate Medical School. Announcement of the Second Year. Sessions of 1883 '84. Nos. 213—215 East 23d Street, New York City.

